# Syndromic surveillance of influenza activity in Sweden: an evaluation of three tools

**DOI:** 10.1017/S0950268814003240

**Published:** 2014-12-04

**Authors:** T. MA, H. ENGLUND, P. BJELKMAR, A. WALLENSTEN, A. HULTH

**Affiliations:** Public Health Agency of Sweden, Solna, Sweden

**Keywords:** Influenza, surveillance system

## Abstract

An evaluation was conducted to determine which syndromic surveillance tools complement traditional surveillance by serving as earlier indicators of influenza activity in Sweden. *Web queries, medical hotline statistics*, and *school absenteeism data* were evaluated against two traditional surveillance tools. Cross-correlation calculations utilized aggregated weekly data for all-age, nationwide activity for four influenza seasons, from 2009/2010 to 2012/2013. The surveillance tool indicative of earlier influenza activity, by way of statistical and visual evidence, was identified. The web query algorithm and medical hotline statistics performed equally well as each other and to the traditional surveillance tools. School absenteeism data were not reliable resources for influenza surveillance. Overall, the syndromic surveillance tools did not perform with enough consistency in season lead nor in earlier timing of the peak week to be considered as early indicators. They do, however, capture incident cases before they have formally entered the primary healthcare system.

## INTRODUCTION

The monitoring of health and disease in populations and over time periods serve important public health functions. Such surveillance elucidates rich descriptive information that can inform both policy and practice. In particular, the surveillance of seasonal influenza results in knowledge about the disease burden in the population as well as the geographical and demographic distribution of cases infected by the virus. This knowledge forms the basis for informed decisions regarding infection control, outbreak management, antiviral distribution, clinical diagnostic guidelines, and vaccination campaigns.

Traditional indicators, such as the proportion of patients presenting at sentinel physician clinics with influenza-like illness (ILI) and the number of laboratory-confirmed cases of influenza can provide a fairly accurate picture of influenza activity patterns as they emerge. However, by solely relying upon data from the healthcare system, the spread of the disease in the community may not be accurately reflected as those who cannot or do not need to seek healthcare services are overlooked.

Syndromic surveillance tools, then, show much promise in complementing the traditional surveillance tools by covering a broader, and perhaps, different segment of the population [1]. Rather than relying upon laboratory confirmation or medical diagnoses, as is the case with traditional surveillance, syndromic surveillance is based on computational methods that use complementary case definitions and statistical thresholds to detect deviations within already existing webs of data, often outside any formal interaction with the healthcare system.

In Sweden, the responsibility for influenza surveillance lies with Folkhälsomyndigheten – the Public Health Agency of Sweden (at the time of analyses, the agency retained its former name as Smittskyddsinstitutet). Embracing both traditional and syndromic surveillance, the agency has established a number of different surveillance tools that are intended to complement each other in providing an overall picture of on-going influenza activity in Sweden. Theoretically, the diversity of surveillance tools used at the agency is designed to monitor varying segments of the population at different stages along the continuum of symptom expression and healthcare-seeking behaviour. However, the true value of the various tools was previously unknown, as no structured evaluation had been performed. Here, we report on the results of a comparison between three syndromic and two traditional surveillance tools used for routine surveillance of nationwide, all-age influenza activity in Sweden. This evaluation was conducted with the primary aim of determining whether, and which, syndromic surveillance tools can provide an added benefit to the traditional surveillance tools by serving as earlier indicators of influenza activity. A secondary aim was to determine whether, and which of, these same tools can complement traditional surveillance by depicting a broader picture of influenza activity in society.

## METHODS

### Data sources

The subsequent two traditional surveillance tools followed by three syndromic surveillance tools were included in the analyses.

#### Sentinel physician reporting of ILI

Every season the agency recruits about 100 physicians working within primary care in Sweden to voluntarily send a weekly electronic report with the number, age, and sex of patients presenting with symptoms of ILI at their clinics during the previous week. Seasonal recruitment is targeted so that there is one sentinel physician practice per 100 000 inhabitants and county. Each week, about 40–45 physician practices submit these data. From the aggregated data of all reports, a proportion measure is calculated by the agency and the following is used as the case definition of influenza activity: ‘the proportion of patients with ILI per 100 000 patients registered with the practice’. Prior to September 2011, this proportion measure utilized a different denominator, and thus, the corresponding case definition for those seasons was ‘the proportion of patients with ILI out of the total number of patients seen’. Both case definitions were included in our analyses for the corresponding seasons in which they were utilized. Our data were corrected for reporting delays.

#### Laboratory reporting of confirmed cases of influenza

Laboratory reporting to the agency is mandatory for confirmed cases of influenza due to A(H1N1)pdm09. Confirmed cases due to other strains are reported on a voluntary basis. In most laboratories (21/25), the reporting is automatic. The remaining laboratories contribute data to the agency manually. The case definition of influenza activity derived from laboratory reports was ‘the number of laboratory-confirmed cases of influenza’.

#### Web queries

Since 2009, estimations by a statistical model based on anonymous web queries submitted to a medical website in Sweden (www.vardguiden.se) have been used in routine influenza surveillance. This web query algorithm represents an estimation of nationwide influenza activity for the previous week [2]. The algorithm, based on 20 types of influenza-related key terms (e.g. fever) with their corresponding weights, is computed every week to capture influenza activity in a manner that mimics the case definition reported by the sentinel reporting tool. The output of this algorithm is ‘the estimated proportion of patients with ILI out of the total number of patients seen by sentinel physicians'.

#### Medical hotline

*The Swedish National Healthcare Guide 1177* is a medical telephone hotline staffed by nurses and owned and operated by county councils across Sweden. For many residents, it serves as the first point of contact with the healthcare system. The primary reason for calling is recorded for all calls to the hotline based on a medical decision system and the medical assessment of the nurse who answered the call. Aggregated data on the primary reason for calling were accessed by the agency for all counties except for Stockholm, Värmland, Sörmland and Norrbotten, where a different system is used. These data cover about 70% of the Swedish population [3]. The following was used as the case definition of influenza activity: ‘the proportion of calls regarding fever out of the total number of calls'.

#### School absenteeism

The agency was given access to aggregated weekday data on school absences due to all-cause illnesses from a private company servicing about 500 schools, ranging from elementary schools to high schools, from around Sweden. This corresponds to about 200 000 children. Schools are notified of an absence on the day of absence via an automated telephone service. ‘The proportion of absent students out of the total number of covered students' was the case definition for this tool.

#### Rationale for selecting the syndromic surveillance sources

The inclusion of the web query tool in our evaluation was a result of its routine use in influenza surveillance at the agency since 2009. This tool was previously evaluated for monitoring of pandemic influenza during the 2009/2010 season, but not for seasonal influenza [4]. Similarly, data on calls to the medical hotline due to influenza-related symptoms has been part of routine influenza surveillance since 2009. Although data from this source had previously been probed for its potential for early event detection and situational awareness of local outbreaks of gastroenteritis [5], the data had yet to be evaluated as a potential tool for influenza surveillance. In another study we modelled influenza cases derived from laboratory reports as a function of all the primary reasons for calling, using data from the medical hotline, and, after interpreting the respective regression coefficients, fever was the symptom that evidenced the most explanatory power (Pär Bjelkmar, personal communication). These results were supported by our qualitative experience. As such, fever was included in the case definition for the medical hotline tool. By contrast to the web query tool and the medical hotline statistics, the agency had yet to use the school absenteeism data for influenza surveillance prior to this evaluation. The Swedish National Board of Health and Welfare has indicated that data on school absenteeism are one of the most important sources for estimating the societal impact of an influenza pandemic [6]. However, previous research has been inconclusive [7–9]. This tool was included to test whether it has potential to contribute to nationwide influenza surveillance in Sweden.

### Evaluation strategy

Our evaluation comprised of a series of retrospective bivariate analyses, which were conducted using statistical software R (http://www.r-project.org/). The first type of comparison evaluated each syndromic surveillance tool, i.e. web query algorithm, school absenteeism data, and medical hotline statistics, against each traditional surveillance tool, i.e. sentinel reporting and laboratory reporting, resulting in six individual comparisons. The second type of comparison evaluated the two traditional surveillance tools against each other, resulting in one comparison between the sentinel reporting tool and the laboratory reporting tool. In total, seven unique comparisons were made, and together they contributed to our overall evaluation. The focus when interpreting the results of our analyses was on temporality of the data, rather than magnitude.

Understanding the progress of influenza activity over the season is important for monitoring of trend. To examine this, we conducted a series of correlation and cross-correlation calculations for each comparison using Pearson's correlation statistic. The tool considered to have the earlier notification ability of the two tools in direct comparison was the one with the most lead, which was determined by the week in which the highest positive correlation occurred, in the series of correlation and cross-correlation calculations up to a period of ±3 weeks.

Additionally, we chose to examine one discrete event within the seasonal pattern, i.e. the peak week. To do so, data derived from each surveillance tool were plotted against each other, yielding a set of two curves superimposed upon one another for each direct comparison. The peak of each influenza curve was inspected for its position (i.e. timing) relative to the other curve. The peak week was chosen because it simultaneously represents the highest population burden of the virus as well as the upcoming decline in influenza incidence. Furthermore, lack of established thresholds precluded our ability to use the discrete events of season start and season end in our evaluation.

Analyses were conducted for aggregated weekly data for all of Sweden across all age groups from week 40 of one year to week 20 of the following year for the following four influenza seasons, where each season was treated separately:

- 2009/2010 (28 September 2009–23 May 2010);
- 2010/2011 (4 October 2010–22 May 2011);
- 2011/2012 (3 October 2011–20 May 2012);
- 2012/2013 (1 October 2012–19 May 2013).

The 2009/2010 season was the first in which the pandemic influenza virus A(H1N1)pdm09 circulated.

## RESULTS

**Fig. 1.**
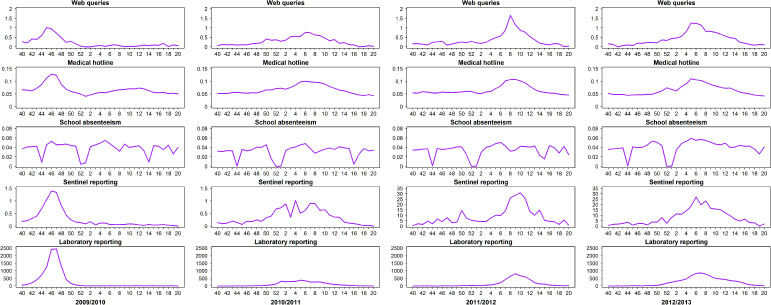
Influenza activity patterns for seasons 2009/2010 to 2012/2013.

Here we present key results. For more detailed results, please refer to Tables 1–3 and Figure 1.

### Comparing syndromic surveillance tools to traditional surveillance tools

The web query algorithm showed a strong positive correlation with both the sentinel reporting and the laboratory reporting tools in all seasons (see Table 1). It demonstrated a 1-week lead in two seasons (2009/2010 and 2011/2012), two instances of concurrence (2010/2011 and 2012/2013) compared to the sentinel reporting tool, one instance of concurrence (2012/2013) compared to the laboratory reporting tool, and one instance of lag by 1 week (2010/2011) compared to the laboratory reporting tool (see Table 1). The peak week, as indicated by the web query tool, occurred 1–2 weeks earlier than indicated in the sentinel reporting and laboratory reporting tool for all seasons except 2010/2011 (see Table 2).

**Table 1.** Comparison of syndromic surveillance tools with traditional surveillance tools

The relative temporal position of the first listed tool in relation to the second (by week)	Concurrent	Lead			Lag		
	Correlation	Cross-correlation –3	Cross-correlation –2	Cross-correlation –1	Cross-correlation +1	Cross-correlation +2	Cross-correlation +3
Web query *vs.* sentinel reporting							
2009/2010	0·935	0·586	0·817	0·951	0·773	0·548	0·344
2010/2011	0·839	0·566	0·72	0·838	0·769	0·765	0·628
2011/2012	0·845	0·605	0·803	0·886	0·632	0·418	0·219
2012/2013	0·943	0·69	0·822	0·925	0·856	0·712	0·567
Web query *vs.* laboratory reporting							
2009/2010	0·874	0·545	0·806	0·944	0·667	0·431	0·246
2010/2011	0·867	0·608	0·73	0·805	0·869	0·823	0·726
2011/2012	0·897	0·659	0·821	0·936	0·712	0·469	0·231
2012/2013	0·908	0·715	0·826	0·888	0·831	0·718	0·566
Medical hotline *vs.* sentinel reporting							
2009/2010	0·914	0·353	0·617	0·836	0·778	0·518	0·24
2010/2011	0·841	0·485	0·643	0·767	0·832	0·781	0·707
2011/2012	0·891	0·606	0·787	0·893	0·752	0·548	0·305
2012/2013	0·927	0·702	0·822	0·922	0·865	0·729	0·568
Medical hotline *vs.* laboratory reporting							
2009/2010	0·914	0·335	0·626	0·867	0·715	0·405	0·134
2010/2011	0·888	0·482	0·637	0·777	0·887	0·861	0·801
2011/2012	0·95	0·641	0·84	0·949	0·808	0·596	0·356
2012/2013	0·957	0·739	0·876	0·952	0·852	0·709	0·547
School absenteeism *vs.* sentinel reporting							
2009/2010	−0·188	−0·1	0·074	0·081	0·245	0·219	0·142
2010/2011	−0·014	0·051	0·076	−0·026	0·084	0·266	0·28
2011/2012	0·243	0·318	0·328	0·238	0·102	0·149	0·075
2012/2013	0·473	0·21	0·298	0·418	0·445	0·389	0·351
School absenteeism *vs.* laboratory reporting							
2009/2010	0·218	−0·15	−0·089	0·139	0·228	0·222	0·163
2010/2011	0·113	−0·008	−0·049	−0·005	0·274	0·314	0·319
2011/2012	0·223	0·348	0·327	0·252	0·228	0·201	0·13
2012/2013	0·451	0·246	0·34	0·419	0·454	0·441	0·394

**Table 2.** Timing of peak week, by tool

Season	Web Query	Medical hotline	School absenteeism	Sentinel reporting	Laboratory reporting
2009/2010	45	46	5	46	47
2010/2011	6	6	6	4	5
2011/2012	8	8	6	10	9
2012/2013	5	5	5	6	7

The medical hotline tool demonstrated a strong positive correlation with both traditional surveillance tools (see Table 1). It captured influenza activity in a timely manner concurrent with the traditional reporting tools. The only exception was during the 2011/2012 season in which the medical hotline tool showed a 1-week lead compared to the sentinel reporting tool (see Table 1). The medical hotline tool did not lag in any season (see Table 1). The peak week from the medical hotline statistics occurred earlier than the two traditional surveillance tools in two seasons, later in one season, and concurrently in the pandemic season (see Table 2).

The school absenteeism data showed a weak positive or, in some cases, negative correlation with both the sentinel reporting and laboratory reporting tools (see Table 1). There was a lag of between 1 and 3 weeks for the majority of the comparisons over all four seasons in relation to the two traditional surveillance tools (see Table 1). However, there were two instances where the school absenteeism data showed a 2- and a 3-week lead ahead of the sentinel reporting and laboratory reporting tools, respectively, and one instance where school absenteeism was concurrent with the sentinel reporting tool (see Table 1). The school absenteeism data indicated the peak week to occur earlier than the other tools in two of the seasons and later in the other two seasons (see Table 2).

### Comparing the two traditional surveillance tools with each other

Overall, the two traditional tools were strongly and positively correlated with each other (see Table 3). They captured influenza activity in a concurrent manner, with neither lead nor lag (see Table 3). An exception was observed in the 2010/2011 season, in which the sentinel reporting tool showed a 1-week lead ahead of the laboratory reporting tool (see Table 3). The peak weeks as indicated by these two traditional surveillance tools occurred 1 week apart, with the laboratory reporting tool ahead in one season and the sentinel reporting tool ahead in the other three seasons (see Table 2).

**Table 3.** Comparison of the two traditional surveillance tools

The relative temporal position of the first listed tool in relation to the second (by week)	Concurrent	Lead			Lag		
	Correlation	Cross-correlation –3	Cross-correlation –2	Cross-correlation –1	Cross-correlation +1	Cross-correlation +2	Cross-correlation +3
Sentinel reporting *vs*. laboratory reporting							
2009/2010	0·976	0·375	0·654	0·898	0·821	0·547	0·294
2010/2011	0·85	0·628	0·754	0·863	0·809	0·695	0·613
2011/2012	0·928	0·439	0·661	0·849	0·856	0·692	0·453
2012/2013	0·964	0·682	0·813	0·923	0·889	0·766	0·618

### Comparing seasonal patterns between all tools

As depicted by the series of influenza curves in Figure 1, the patterns for three seasons (2009/2010, 2011/2012, 2012/2013) for all tools except school absenteeism data show a major increase in the number of cases until a peak is reached, after which the seasons experienced a gradual decline in influenza activity. The 2010/2011 season is unique in that it was characterized by the co-circulation of both A(H1N1)pdm09 and influenza B, each of which had a separate peak. This makes the overall peak from the aggregated data, as depicted in Figure 1, less pronounced. In that same season, influenza B was the dominant strain, which also contributed to a less pronounced peak over the season as cases of that strain were detected at a lower rate for a longer period of time.

## DISCUSSION

The Public Health Agency of Sweden's influenza surveillance is designed to provide a comprehensive picture of ongoing and overall seasonal influenza activity across the nation, by use of both traditional and syndromic surveillance tools. The functioning of different tools used within surveillance was evaluated via a series of retrospective bivariate comparisons, which considered (1) each syndromic surveillance tool to each traditional surveillance tool, and (2) the two traditional surveillance tools to each other.

Such comparisons would ideally be based around a gold standard – a reference point around which to articulate differences, changes, or improvements. However, in the context of influenza surveillance, there is no gold standard as the available tools do not capture the entire range of influenza activity. Surveillance tools are continually iterated to monitor and track the natural progression and spread of a disease. Thus, they provide estimates at best and, even those considered the most accurate and relied upon most, are not perfect reflections of reality. Cognisant of this limitation, but lacking other reference options, we referred to the traditional surveillance tools as references in order to facilitate the comparisons in this evaluation. However, the results of our evaluation remain working conclusions and relative judgements, and not absolute statements that rank the various surveillance tools in a hierarchy of accuracy.

The strong performance of the web query algorithm is no surprise as it was based on trained statistical models with a specific goal to mimic traditional surveillance. This is in contrast to the other two syndromic surveillance tools, which were both based on data without any further statistical refinement. The results of the web query tool are available early Monday mornings, in comparison to the traditional tools for which data are collated before Wednesday afternoons. Because of this, the web query tool, in effect, has a certain lead over the traditional surveillance tools, although it did not show a marked lead in our analysis. The most well-known web query-based influenza tool is undoubtedly Google Flu Trends (GFT) [10]. Evaluations of GFT estimations for the United States showed that the algorithm greatly overestimated the magnitude of the influenza season 2012/2013 [11, 12]. Based on a comparison of 10 years of data for three different geographical levels, the conclusion was that GFT cannot provide reliable surveillance data for influenza surveillance [12]. However, the web query data used at the agency originate from a designated health portal, which may explain the high correlation between this tool and the traditional influenza surveillance tools. Our results show that this tool is most reliable for both pandemic and seasonal influenza surveillance. The presented results are congruent with a previous evaluation of similar aims, which found a significant correlation between data mined from a different web source, i.e. blog posts, and reported data on ILI during the 2008/2009 influenza season in the United States [13].

Like the web query algorithm, the medical hotline statistics performed strongly as a syndromic surveillance tool for monitoring influenza patterns. This may be due to it receiving a high volume of calls per day and its high visibility and uptake in the general Swedish population. Moreover, this tool utilized a symptom that was specifically selected based on preliminary analyses demonstrating evidence of its correlation with laboratory-confirmed cases of influenza. The training and experience of the nurses who answer calls and subsequently record the primary reason for calling minimized the chance of a misclassification of symptoms. Taken together, these reasons may provide explanations for the success of the medical hotline tool as a surveillance resource that can accurately capture influenza activity. The success of this tool is in line with previous experience in Canada [14] and the UK [15].

School absenteeism data performed inconsistently and inaccurately in the current evaluation. This tool demonstrated a weak and sometimes negative correlation with traditional surveillance tools, a tendency to lag, and inconsistent estimations of the peak week relative to the two traditional tools. Possible reasons for this is that the data, in their current form, represent absences due to all illnesses and that the students are not a representative sample of the Swedish population when it comes to age and geographical distribution. Another reason is that no reporting is done during school holidays, which means that the influenza curves for this tool have shapes that are very different from those of traditional influenza surveillance tools. Together, the weak correlation and the tendency to lag undermine the utility of using school absenteeism data in its current form as a surveillance tool for influenza activity in Sweden. In a British study of influenza activity two seasons prior to and during the 2009 influenza A(H1N1) pandemic, school absenteeism data *did* perform consistently with traditional surveillance [9]. Possible reasons for divergent findings between our study and the British study may be due to different case definitions and population coverage at the local *vs.* the national level. Despite the accuracy of school absenteeism data in the British study, the authors conclude that their utility for early detection still remains to be determined [9]. Because data on children are important for the understanding of communicable disease dynamics, ways to improve the usefulness of this tool should be investigated, for example, by developing daily absenteeism reporting systems as well as baselines and thresholds.

In regard to our primary aim of evaluating the syndromic surveillance tools for potential to serve as earlier indicators of influenza activity, the three syndromic surveillance tools under scrutiny did not perform with enough consistency in season lead nor in earlier timing of the peak week to be considered as an early notification system. Regarding our secondary aim of evaluating whether the syndromic surveillance tools cover a broader spectrum of the population, taking into account those at different stages of symptom expression and those with different healthcare-seeking behaviours, all three syndromic surveillance tools have the potential to capture the incidence of influenza of those who have yet to formally enter the primary healthcare system and consult in person with a physician. However, it was only the web queries and the medical hotline that showed strong correlation with traditional surveillance. Furthermore, it should be noted that syndromic surveillance tools accrue additional benefits above and beyond those of traditional surveillance tools in that they are less resource-dependent and more automatic.

The two traditional surveillance tools produced highly similar outputs in tracking influenza activity over the entire season and in peak week occurrence. The main difference between them is in the level of additional details concerning the characteristics of circulating influenza strains. For this information, the laboratory reporting tool yields an added benefit as it can also provide information on virological subtypes. Since the sentinel reporting tool contributes no additional benefit over a surveillance system that hypothetically uses only the laboratory reporting, web query, and medical hotline tools, it could be discontinued without much disruption to routine influenza surveillance. Additional arguments for discontinuing the sentinel reporting tool include that participation of sentinel physicians is often below par due to the additional burden to their workload. Inadequate participation by sentinel physicians may lead to an imbalanced geographical representation of data on influenza activity. By contrast, retaining the sentinel reporting tool may be preferred as it indirectly provides unique information on the resource burden on primary care and covers the segment of the population in which people formally enter the healthcare system by consulting in person with a physician. Additionally, the sentinel reporting tool is one of the most well established methods for influenza surveillance in the world.

A systematic evaluation of the various tools that comprise Sweden's routine influenza surveillance system provides evidence-based information for the agency to influence policy and practice at the national level. It also keeps Sweden accountable to the global surveillance networks to which it contributes data, including The European Surveillance System of the European Centre for Disease Prevention and Control, and FluNet of the World Health Organization.

## CONCLUSIONS

When comparing the syndromic surveillance tools with the traditional surveillance tools, we found that the web query algorithm and the medical hotline statistics robustly reflected influenza activity patterns as indicated by their strong positive correlation with traditional surveillance tools, as well as by their ability to capture both the progress of influenza activity over the season and the peak week with concurrence or lead relative to the traditional surveillance tools. Given their strong performance in the evaluations, both the web query algorithm and the medical hotline statistics are considered to be on a par with each other and with both traditional surveillance tools, providing the agency with two additional and validated resources to perform its routine influenza monitoring functions for Sweden at low additional cost. School absenteeism data cannot, in its current form, serve as a surveillance tool for influenza. More detailed evaluations covering a longer time span and stratified per age group and geography are warranted before it can be concluded that the web query tool and the medical hotline tool have potential to replace the resource-dependent sentinel reporting tool.
